# Shelf life of air and modified atmosphere-packaged fresh tilapia (*Oreochromis niloticus*) fillets stored under chilled and superchilled conditions

**DOI:** 10.1002/fsn3.18

**Published:** 2012-12-26

**Authors:** Odoli Cyprian, Hélène L Lauzon, Ragnar Jóhannsson, Kolbrún Sveinsdóttir, Sigurjón Arason, Emilía Martinsdóttir

**Affiliations:** 1 Kenya Marine and Fisheries Research InstituteMombasa, Kenya; 2 Faculty of Food Science and Nutrition, University of IcelandSæmundargötu 2, Reykjavík, Iceland; 3 Matís ohf./Icelandic Food and Biotech R&D Vínlandsleið 12Reykjavik, Iceland

**Keywords:** Air packaged, modified atmosphere, shelf life, superchilling, tilapia fillets

## Abstract

Optimal packaging and storage conditions for fresh tilapia fillets were established by evaluating sensory and microbiological changes, as well as monitoring physicochemical properties. Nile tilapia (*Oreochromis niloticus*) farmed in recirculation aquaculture system was filleted, deskinned, and packaged in air and 50% CO_2_/50% N_2_ prior to chilling and superchilling storage at 1°C and −1°C. Sensory analysis of cooked samples revealed a shelf life of 13–15 days for air-packaged fillets during storage at 1°C and 20 days at −1°C. At the end of shelf life in air-packaged fillets, total viable counts (TVC) and pseudomonads counts reached log 8 colony-forming units (CFU) g^−1^. In 50% CO_2_/50% N_2_-packaged fillets, the lag phase and generation time of bacteria were extended and recorded counts were below the limit for consumption (<log 8 CFU g^−1^) after 23 days of storage at both 1°C and −1°C. However, modified atmosphere (MA) packaging negatively affected color characteristics of the fillets soon after packaging (day 6). Color is an important indicator of tilapia fillets quality and a major factor in influencing retail purchase decisions. In view of that, air packaged at −1°C storage temperature was the optimal condition for fresh tilapia fillets. Total volatile basic nitrogen (TVB-N) and trimethylamine (TMA) were not good indicators of spoilage of tilapia fillets in this study.

## Practical Applications

The rapidly developing retail market of tilapia fillets and demand for fresh products emphasize the need for prolonged shelf life of fresh fillets. In this study, different storage conditions for fresh fillets are assessed to establish optimal storage conditions. The study shows that air packaged in a high-barrier film bag combined with low-temperature storage maintains superior fillet quality longer and prolonged the shelf life of fillets stored at −1°C by 5–7 days as compared with storage at +1°C.

## Introduction

With increased awareness, the consumers are progressively demanding high food quality and have corresponding expectations that quality will be maintained at a high level during the period between production and consumption (Fisheries and Aquaculture Department 2002). However, the shelf life of fresh fishery products is usually limited by microbial activities that are influenced most importantly by storage temperature (Huss 1995; Simpson et al. 2003). Typical shelf life under icing storage conditions ranges from 6 to 20 days (Martinsdottir et al. 2001; Cyprian et al. 2008) depending on species, harvest location, and season, and short shelf life can result in heavy economic loss (Reddy et al. 1995; Sivertsvik et al. 2002). This has made packaging an integral part of the food industry, as in addition to preservation function, it has several other important roles to play in delivering safe, wholesome, and attractive foods to the market (Kilcast and Subramaniam 2000).

Modified atmosphere packaging (MAP) of fishery products has been shown to inhibit the normal spoilage flora and increase shelf life significantly at refrigerated temperatures (Sivertsvik et al. 2002). The application of this technology to foods has become increasingly more available in recent years (Sveinsdottir et al. 2010), as food manufacturers have attempted to meet consumer demands. Studies have shown MAP to perform an effective synergy with superchilling in prolonging the shelf life of fresh cod (*Gadus morhua*) loins (Wang et al. 2008; Lauzon et al. 2009) and Atlantic salmon (*Salmo salar*) fillets (Sivertsvik et al. 2003; Duun and Rustad 2008). Duun and Rustad (2008) reported superchilled salmon fillets stored at −2°C in combination with MAP to have maintained good quality with negligible microbial growth for more than 24 days based on both sensory and microbial analyses, whereas ice-chilled reference fillets maintained good quality up to 17 days.

Nevertheless packaging technologists should be aware of a major concern limiting the development of MAP for this product group, namely *Clostridium botulinum. Clostridium botulinum* (anaerobic) has often been reported in fresh seafood products that have been packaged under regimes designed to limit the availability of oxygen (Aberoumand 2010). Specifically, *C. botulinum* type E is of most concern in this type of packaging as it is a naturally occurring aquatic organism and grows at refrigeration temperature as low as 2.9°C (Conner et al. 1989). To ensure safety and success of MAP, packaged products should be kept at refrigeration temperature below 2.9°C.

MA packaging of tilapia (*Oreochromis niloticus*) fillets in 75% CO_2_/25% N_2_ stored at 4°C was previously evaluated and promising results reported (Reddy et al. 1995). This study aimed at investigating the combined effect of MA packaging (50% CO_2_/50% N_2_) and superchilling on the sensory, microbiological, and chemical changes during storage of fresh tilapia fillets.

## Materials and Methods

### Experimental design

Tilapia (*O. niloticus*) used for the study was farmed in recirculation aquaculture system (RAS) at Straumfraedihus Keldnaholti in Reykjavik, Iceland. A total of 340 fish weighing between 450 and 700 g were harvested, bled, and transported iced to the laboratory within 3 h. On arrival, fish was hand filleted and deskinned, and 24 fillets (control) were immediately used for sensory, microbiological, and chemical analyses. The rest was kept chilled at 0°C while packaging. Four to five deskinned fillets weighing 250–290 g (altogether) were placed with skin side up on a preweighed foam tray (expanded polystyrene, Linstar E 39-34, LINPAC, Plastprent, Iceland) with a built-in absorption mat and packaged with a high-barrier film bag (250 × 400 × 0.120 mm coextruded PA/PE, Plastprent hf., Iceland). Four groups of 40 packs each were prepared (160 packs altogether) based on atmospheric and storage conditions.

In the first two groups (T1 and T2), each bag was sealed containing air using a vacuum packaging machine (HENKOVAC Heavy duty 2000, Hertogenbosch, the Netherlands) equipped with a built-in vacuum pump and gas flush. The third and fourth groups (T3 and T4) were evacuated and packaged under MA (50% CO_2_/50% N_2_). CO_2_ and N_2_ were mixed using a gas mixer MAP Mix 9000 (PBI-Dansensor, Ringsted, Denmark). After packaging, T1 and T3 packages were stored at 1°C, whereas, T2 and T4 were stored at −1°C throughout the study. The description of groups T1, T2, T3, and T4 is given in Table 1.

**Table 1:** Definition of treatment groups studied and sampling scheme

Treatment codes	Atmosphere at packaging	Storage temperature	Number of packs (*n*)	Sampling days
Control	Not packaged	Iced		0
T1	Air	1°C	40	2, 6, 9, 13, 16, 20
T2	Air	−1°C	40	2, 6, 9, 13, 16, 20
T3	50% CO_2_/50% N_2_	1°C	40	2, 6, 9, 13, 16, 20, 23
T4	50% CO_2_/50% N_2_	−1°C	40	2, 6, 9, 13, 16, 20, 23

On every sampling day, five packs per treatment were taken (20 altogether). The five packs were analyzed for headspace gas composition, and thereafter, two packs were used for microbial and chemical analysis, with the remaining three packs used for exudate measurements and sensory evaluation (raw and cooked fillets).

### Gas analysis

The headspace gas composition in air and MA packaged was determined upon packaging and on every sampling in five packs for each treatment, using the gas analyzer (CheckMate 9900 Analyzer, PBI-Dansensor, Ringsted, Denmark). The concentrations of sampled packages with respect to treatments were averaged (*n* = 5).

### Sensory evaluation of raw and cooked fillets

Evaluation of raw fillets was done using a Quality Index Method (QIM) scheme developed for deskinned tilapia fillets (Table 2) according to the methodology described earlier by Martinsdottir et al. (2001) and Hyldig et al. (2007). The QIM scheme was based on significant, well-defined characteristics of appearance, odor, and texture attributes changing through storage time. All observations of tilapia fillets were conducted under standardized conditions following the general guidance for the design of test room and testing conditions described in ISO 8589 (1988). On every sampling day, three fillets were taken randomly from three different packs for each group and placed on a clean table. Each fillet was blind coded with a number consisting of three digits that did not indicate storage conditions. Eight to twelve trained panelists, all employees of Matis ohf with good use of vocabulary and knowledge of the fish species, individually evaluated changes in color, mucus, texture, and odor. The selection and training of the panel members on tilapia attributes were carried out during three training sessions (preliminary study) according to international standards ISO 8586 (ISO 1993), which include detection and recognition of tastes and odors, use of scales, and the development of descriptors. A score from 0 to 3 demerit points was given for every attribute evaluated (Table 2).

**Table 2:** A modified Quality Index Method scheme developed from preliminary scheme for deskinned tilapia fillets (O. Cyprian et al. unpubl. data)

Quality parameter	Description	Demerit point
Skin side		
Color^1^	Dark red, red brown	0
	Red brown, lighter color	1
	Light brown	2
Flesh		
Color, loin	Light, beige, trace of red or bluish	0
	A little darker color, a little brownish or grayish	1
	Grayish, brownish, yellowish	2
Color, flap	Bluish, transparent	0
	Light, milky color	1
	Grayish or brownish	2
Mucus	Fresh shiny texture, thin clear mucus	0
	Trace of mucus, a little thick	1
	Milky or greenish mucus	2
Texture	Firm	0
	Rather soft	1
	Soft	2
Odor	Fresh, neutral	0
	Seaweed, marine, grass	1
	Sour milk, silage	2
	Acetic, putrid	3
Quality index (0–13)		

1 Lateral/longitudinal stripes at the middle of the loin.

Sensory evaluation of cooked tilapia fillets was performed in parallel to the QIM evaluation. Fillet loins from the three packs for each group were cut into pieces of about 4–5 cm long and 3–4 cm wide. The pieces were placed in aluminum boxes coded with three-digit random numbers and cooked in a preheated electric oven Convostar (Convotherm GmbH, Eglfing, Germany) with circulation air and steam at 95–100°C for 6 min. Eight to twelve panelists trained in recognition of sensory characteristics of the samples and to describe the intensity of each attribute using an unstructured scale from 0% to 100% (Stone and Sidel 1985) were served with cooked samples. Each panelist evaluated duplicates of samples in a random order for each group based on sensory vocabulary of cooked tilapia (Table 3). A computerized system (FIZZ, Version 2.0, 1994–2000, Biosystèmes, France) was used for data recording.

**Table 3:** Sensory vocabulary for cooked tilapia

Short name	Sensory attribute	Description of attribute
Odor		
O-Boilpot	Boiled potatoes	Whole newly boiled potatoes
O-Boilmilk	Boiled milk	Hot milk, fruity odor
O-Earthy	Earth	Fresh earth
O-Musty	Musty	Moldy
O-Rancid	Rancid	Rancidity
Appearance		
A-Color (T)	Light/dark color	Light, white or dark, yellowish color
A-Color (U)	Dark brown/light gray	Dark brownish or light gray color
A-BlackThr	Black threads	Black threads in flesh
Flavor		
F-Arctic charr	Arctic charr	Arctic charr, new trout
F-Sweet	Sweet	Typical sweet flavor of fresh fish
F-Metallic	Metallic	Metallic flavor
F-Earthy	Earthy	Fresh earth
F-Musty	Musty	Moldy flavor
F-Sour	Sour	Sour taste, spoilage sour
F-Pungent	Pungent	Pungent flavor, bitter
F-Rancid	Rancid	Rancidity, cod liver oil, reminds of paint or solvent/thinner
F-Spoilage	Spoilage	Spoilage, queasy sweet flavor
Texture		
T-Flakes	Flakiness	Fish portion slides into flakes when pressed with a fork
T-Soft	Firm/soft	How firm or soft the fish is during the first bite
T-Juicy	Dry/juicy	When chewed, dry: pulls liquid from mouth; juicy: gives liquid
T-Fibers	Fiber	Roughness of muscle fiber
T-Mushy	Mushy	Mushiness when chewed (mushy texture)
T-Sticky	Sticky	Glues together teeth when biting the fish

### Microbiological analysis

Fillets were sampled aseptically and analyzed in duplicate for each pack for two packages per treatment. Fillets were minced in a sterile blender, assessing two pooled fillets for each replicate sample (*n* = 2). Minced flesh (20 g) was mixed with 180 g of chilled Maximum Recovery Diluent (MRD, Oxoid, U.K.) in a stomacher for 1 min. Successive 10-fold dilutions were done as required. Total viable counts (TVC) were obtained by spread plating of aliquots on iron agar (IA) modified from Gram et al. (1987), containing 1% NaCl (no overlay), followed by aerobic incubation at 17°C for 5 days. Counts of H_2_S-producing bacteria were also evaluated by counting black colonies on IA. Presumptive *Pseudomonas* counts (22°C, 3 days) were obtained using the modified cephaloridine–fucidin–cetrimide (CFC) agar as described by Stanbridge and Board (1994). *Pseudomonas* agar base (Oxoid) with CFC Selective Agar Supplement (Oxoid) was used. *Pseudomonas* spp. form pink colonies on this medium.

### pH measurement

Measurements of pH were done using 5 g of minced fish mixed with 5 mL of deionized water. The pH meter Radiometer PHM 80 was calibrated using the buffer solutions of pH 7.00 ± 0.01 and 4.01 ± 0.01 (25°C) (Radiometer Analytical A/S, Bagsvaerd, Denmark). All measurements were done in duplicate per package (for two packs per group) and results presented as an average.

### Assessment of exudate in the packages

Exudate accumulation in the packages during storage was measured gravimetrically using three packs per treatments (*n* = 3). The mass of the exudate (g) was divided by the initial mass of the fish product (g) and reported as a percentage of drip loss.

### Total volatile basic nitrogen and trimethylamine

Duplicates of fillet mince for each treatment that remained after microbiological analyses were used for total volatile basic nitrogen (TVB-N) and trimethylamine (TMA) analysis. The method of Malle and Tao (1987) was used. TVB-N was measured by steam distillation (Struer TVN distillatory, STRUERS, Copenhagen, Denmark) and titration, after extracting the fish muscle with 7.5% aqueous trichloroacetic acid (TCA) solution. The distilled TVB-N was collected in boric acid solution and then titrated with sulfuric acid solution. TMA was measured in TCA extract by adding 20 mL of 35% formaldehyde, an alkaline binding mono- and diamine, TMA being the only volatile and measurable amine.

### Data analysis

The mean values of measured parameters were plotted separately against storage time using Microsoft Excel (2007). Multivariate comparison of different sensory attributes and samples was performed with principal component analysis (PCA) on mean sensory attribute values using full cross-validation in the statistical program Unscrambler ® (Version 8.0 CAMO, Trondheim, Norway). Analysis of variance (ANOVA) of the results was performed in the statistical program NCSS 2000 (NCSS, UT). The program (ANOVA) calculates multiple comparisons using Duncan's test to determine if sample groups are different. Significance of difference was defined at the 5% level.

## Results and Discussion

### Headspace gas composition

**Figure 1:**
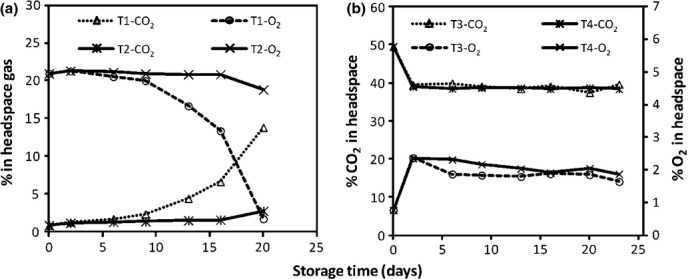
Gas composition changes in headspace of air (a) and modified atmosphere (MA) (b) packaged tilapia fillets during storage at 1°C and −1°C (average values for packs, *n* = 5).

The gas product ratio in the packages was 5:1. At packaging, air packaged (T1 and T2) had approximately 21% oxygen and 0% carbon dioxide as shown in Figure 1a. Oxygen levels in all the groups showed similar trends of decline with storage time as reported elsewhere (Reddy et al. 1995; Hovda et al. 2007). The largest decline occurred in air-packaged samples stored at 1°C (T1) which corresponded with the occurrence of high bacterial numbers (Fig. 4) suggesting that the oxygen was being consumed during microbial metabolism. At −1°C, oxygen consumption by bacteria was delayed and occurred to a lesser extent, being first observed after 16 days of storage coinciding with a bacterial load of about log 6 colony-forming units (CFU) g^−1^. Similar trends in the opposed direction were observed with CO_2_ composition in the headspace of air-packaged fillets, where CO_2_ increased as a secondary product of microbial metabolism (Hovda et al. 2007).

Initially, the headspace of MA-packaged fillets (T3 and T4) had a low oxygen level (<1%), which was observed to be higher on subsequent sampling on day 2 (>2%) and thereafter gradually declined (Fig. 1b). This may be explained by the presence of oxygen in the muscle being released to the headspace upon dissolution of carbon dioxide in the muscle water phase after packaging and gas equilibrium was reached. In packages stored at 1°C, the oxygen drop was more pronounced than at −1°C due to a faster microbial proliferation. However, O_2_ did not decrease below the initial 1% as less microbial growth was reported in MA-packaged groups. On the other hand, CO_2_ reduced from 50% at packaging to around 40% during early storage as it dissolved into fillets due to its high solubility in water and fat at low temperature (Soccol and Oetterer 2003).

### Sensory evaluation using QIM (raw fillets)

**Figure 2:**
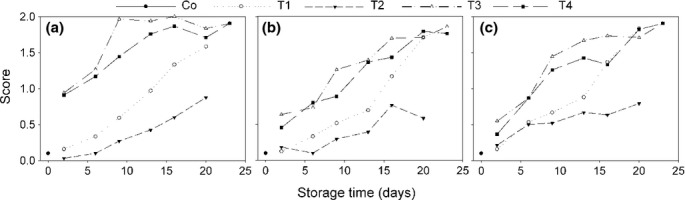
Average scores (three fillets from three packs, *n* = 3) of the appearance parameters as assessed by Quality Index Method (QIM) scheme for deskinned tilapia fillets during storage at 1°C and −1°C. (a) Flesh color from skin side; (b) flesh color from loin side; and (c) color of belly flap.

The QIM scheme is constructed in that fish evaluated shortly after catch should be scored low and subsequently increases with storage time reaching close to maximum score at the end of shelf life (Martinsdottir et al. 2001). The first change observed in air- and MA-packaged tilapia fillets was deterioration in muscle appearance on day 2 of storage at both 1°C and −1°C. At the beginning of storage trial, tilapia fillets were dark red in color on the skin side, but the backbone side had a light appearance with trace of red color or hue (Table 2 and Fig. 2a and b). According to the appearance parameters, color faded soon after packaging under MA. On day 2, it was characterized by red brown and light brown on the skin and backbone sides, but the color progressed to light brown and grayish brown, respectively, toward the end of storage time. As from day 2, MA treatments recorded significantly higher scores (*P* < 0.05) for the appearance parameters compared with air packaged except for T1 toward end of storage time (day 16 and 20). Skin discoloration of some MA-treated fish samples has previously been reported (Stansby and Griffiths 1935; Lauzon et al. 2002) and is due to denaturation of muscle and pigment proteins. The discoloration process was slower in air packaged as fillets retained the dark red color characteristic longer, especially with storage at −1°C which maintained a stable color until the end of storage time at day 20. This could be explained by the maintenance of the oxymyoglobin pigment in the muscle in presence of oxygen, being more soluble under superchilled conditions (Sivertsvik et al. 2003; Ando et al. 2004; Ando et al. 2005).

### Sensory evaluation of cooked tilapia fillets

**Figure 3:**
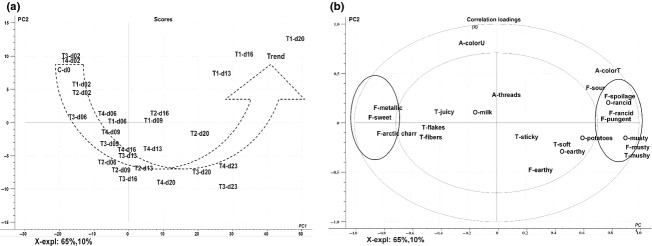
Scores (a) and correlation loadings (b) describing sensory quality of cooked tilapia fillets as evaluated by a trained sensory panel. Principal component 1 (PC1) (65%) versus PC2 (10%). d, storage days; T1, T2, T3, and T4, treatment groups; F, flavor; O, odor; A, appearance; T, texture.

Figure 3 shows how sample groups were described by sensory attributes of cooked tilapia fillets with storage time. Sensory quality attributes detected at the beginning of storage time (sweet, metallic, and arctic charr flavor) considered freshness attributes were positioned left along the first principal component (PC1), whereas attributes detected at the end of storage time (rancid, spoilage, musty, and pungent flavor) were positioned to the right and considered spoilage attributes (Sveinsdottir et al. 2002; Cyprian et al. 2008). The samples varied mainly with regard to differences in flavor and odor attributes along PC1, explaining 65% of the variation between the sample groups. Samples also varied with regard to differences in appearance and texture attributes along the second principal component (PC2), explaining 10% of the variation. The main difference occurred with storage time, as the sample groups are located to the left side at the beginning of storage but on the right side toward end of storage time (Fig. 3a).

As time progressed, the first noticeable difference was on day 9 when rancid flavor was noticed in T1, as well as other negative parameters from day 13 (Table 4). End of shelf life is usually determined when spoilage-related sensory attributes become evident and most panelists detect them. The average score of above 20 (on the scale 0–100) for these spoilage attributes has been applied by various authors as an indication that fish sample is approaching the end of shelf life (Sveinsdottir et al. 2002; Magnússon et al. 2006; Bonilla et al. 2007; Cyprian et al. 2008). Air-packaged fillets stored at 1°C (T1) showed first spoilage characteristics on day 13. On day 16 and day 20, groups T2, T3, and T4 also showed signs of spoilage. However, the scores for spoilage attributes were below 20 on day 23 for groups T3 and T4. On the other hand, a part of the panel could not taste T1 samples due to strong spoilage odor indicating that the group (T1) was no longer fit for human consumption on that day. Similar observation was noted by Sveinsdottir et al. (2002) who reported salmon to be unfit for consumption when part of the panel did not taste samples due to spoilage odor. T2 and T3 recorded spoilage attributes scores close to 20 on day 20 and 23, respectively, implying the groups may have been approaching the end of shelf life.

**Table 4:** Mean sensory scores (scale 0–100) of odor, flavor, and appearance/texture attributes for cooked tilapia fillets

Storage time (days)	Group	Odor		Appearance/texture		Flavor			
		
		Musty	Rancid	Color T	Juicy	Arctic charr	Sweet	Rancid	Spoilage
0	Co	23	3	28	63	33	33	4	3
2	T1	28	2	30	60	37	36	2	2
	T2	25	2	34	63	37	35	2	2
	T3	24	3	34	62	34	35	2	2
	T4	23	2	29	64	36	35	2	2
	*P*-value	0.905	0.699	0.347	0.775	0.737	0.999	0.997	0.990
6	T1	26	9	31	59	34	30	7	7
	T2	26	4	28	60	33	33	6	4
	T3	20	3	27	52	34	33	4	3
	T4	25	6	26	57	32	31	7	5
	*P*-value	0.252	0.080	0.636	0.095	0.937	0.765	0.483	0.580
9	T1	27	8	34	59	32	25	12^a^	6
	T2	24	5	32	60	38	30	6^b^	3
	T3	23	6	26	57	36	29	4^b^	6
	T4	25	5	30	55	38	27	4^b^	1
	*P*-value	0.586	0.554	0.320	0.512	0.075	0.479	**0.020**	0.130
13	T1	31^a^	15^a^	45^a^	53	30	22	24^a^	20^a^
	T2	30^a^	4^b^	31^b^	61	32	24	5^b^	9^b^
	T3	25^b^	5^b^	31^b^	56	24	25	7^b^	5^b^
	T4	24^b^	6^b^	34^b^	54	20	23	12^b^	8^b^
	*P*-value	**0.004**	**<0.001**	**<0.001**	0.122	0.254	0.804	**<0.001**	**0.002**
16	T1	29	20^a^	44^a^	55	27^a^	22^a^	22^a^	26^a^
	T2	24	12^a^	30^b^	60	34^b^	23^a^	13^b^	12^b^
	T3	25	8^b^	25^b^	57	36^b^	30^b^	10^b^	8^b^
	T4	27	7^b^	27^b^	54	34^b^	29^b^	10^b^	7^b^
	*P*-value	0.328	**0.014**	**<0.001**	0.450	**0.002**	**0.006**	**0.024**	**<0.001**
20	T1	32	25^a^	47^a^	55^a^	28	14^a^	25^a^	36^a^
	T2	26	14^b^	32^b^	64^b^	34	24^b^	18	18^b^
	T3	28	14^b^	30^b^	55^a^	33	22^b^	18	19^b^
	T4	28	10^b^	28^b^	58	32	25^b^	12^b^	12^b^
	*P*-value	0.368	**0.004**	**<0.001**	**0.042**	0.075	**0.006**	**0.049**	**<0.001**
23	T3	29	13	28^a^	58	26	21	18	14
	T4	34	14	37^b^	62	28	20	19	18
	*P*-value	0.377	0.893	**0.038**	0.646	0.588	0.432	0.472	0.256

Data within the same column with respect to storage time (days) with different letters are significantly (*P* < 0.05 in bold) different.

### Microbiological analyses

**Figure 4:**
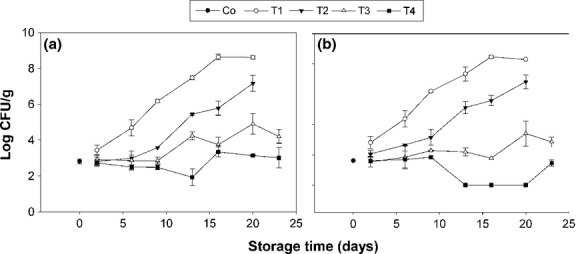
Changes in total viable counts on iron agar (a) and counts of presumptive pseudomonads (b) in air- and modified atmosphere (MA)-packaged tilapia fillets during storage at 1°C and −1°C (duplicates per pack for two packs, *n* = 2).

Huss (1995) reported *Pseudomonas* spp. to be the specific spoilage bacteria of iced stored tropical freshwater fish. Figure 4A and B shows the growth curves for TVC and pseudomonads in air- and MA-packaged tilapia fillets stored at 1°C and at −1°C. TVC maximum limit of log 8 CFU g^−1^ has previously been used in tilapia as the limit for human consumption (Reddy et al. 1995; Waliszewski and Avalos 2001). In this study, TVC and *Pseudomonas* spp. had reached levels greater than the above-reported consumption limit for air-packaged fillets during storage at 1°C (T1) on day 16 (Fig. 4a and b) indicating that they had surpassed the consumption limit. No further bacterial growth was observed after day 16. This observation could also be attributed to change in headspace gas composition (Fig. 1a) whereby the exponential aerobic growth resulted to high oxygen consumption and an increase in CO_2_ as a secondary product of metabolism whose accumulation may have restricted further growth (Adams and Moss 2008). Pseudomonads were prevailing among the spoilage flora of tilapia fillets. A count of log 7.3 CFU g^−1^ was reached at onset of sensory spoilage in T1 samples (day 13). This is close to the reported pseudomonad spoilage level of log 7 CFU g^−1^ in refrigerated cod products (Gospavic et al. 2011). In air-packaged fillets stored at −1°C, TVC and *Pseudomonas* spp. counts were close to log 7 CFU g^−1^ on day 20, at the onset of spoilage for T2. The low count in air-packaged fillets during storage at −1°C compared with 1°C is due to the effective delay of bacterial growth during storage at superchilling conditions (Huss 1995; Church 1998). MA groups recorded extended lag phase during early storage days and aerobic counts of less than log 4 CFU g^−1^ were seen up to day 23. This indicates that aerobic counts were too low to cause microbial deterioration in MA-packaged fillets (Fig. 4), in agreement with the sensory data reported. The delayed growth and low counts of aerobic microorganisms in MA-packaged fillets can be attributed mainly to CO_2_ inhibition (Sivertsvik et al. 2002) and the presence of low level O_2_ (Reddy and Armstrong 1992) but also to low storage temperature (Huss 1995) used in this study. Throughout the storage time, low counts (<log 3.0 CFU g^−1^) of H_2_S-producing bacteria, a recognized spoilage bacterial group including several *Shewanella spp*. (Vogel et al. 2005), were enumerated in all the groups (data not provided), which was attributed to the poor growth of these bacteria at low flesh pH (Table 5 as previous reported by Sivertsvik et al. (2003) and Gram and Huss (1996) as well as probable competition with pseudomonads (Gram and Melchiorsen 1992).

**Table 5:** pH changes in air- and modified atmosphere (MA)-packaged tilapia fillets during storage at 1°C and −1°C (average values of duplicates per pack for two packs and SD shown, *n* = 2). Data within the same column with different letters are significantly (*P* < 0.05 in bold) different

Group	Day							

	0	2	6	9	13	16	20	23
Control	6.5±0	–	–	–	–	–	–	–
T1		6.5±0	6.6±0	6.6±0.1	6.5±0.1	6.6±0.3	6.7±0	–
T2		6.6±0.1	6.7±0.1	6.4±0	6.6±0.1	6.4±0	6.5±0.1	–
T3		6.4±0^c^	6.5±0.1	6.4±0	6.5±0.1	6.4±0.1	6.5±0.2	6.5±0
T4		6.5±0	6.5±0.1	6.5±0	6.4±0	6.4±0.2	6.5±0.1	6.5±0.1
*P*-value		**0.049**	0.111	0.138	0.242	0.501	0.150	0.422

c Data within the same column in respect to treatment (group) with different letters (superscript) are significantly (*P* < 0.05 in bold) different.

### pH changes and drip loss

**Figure 5:**
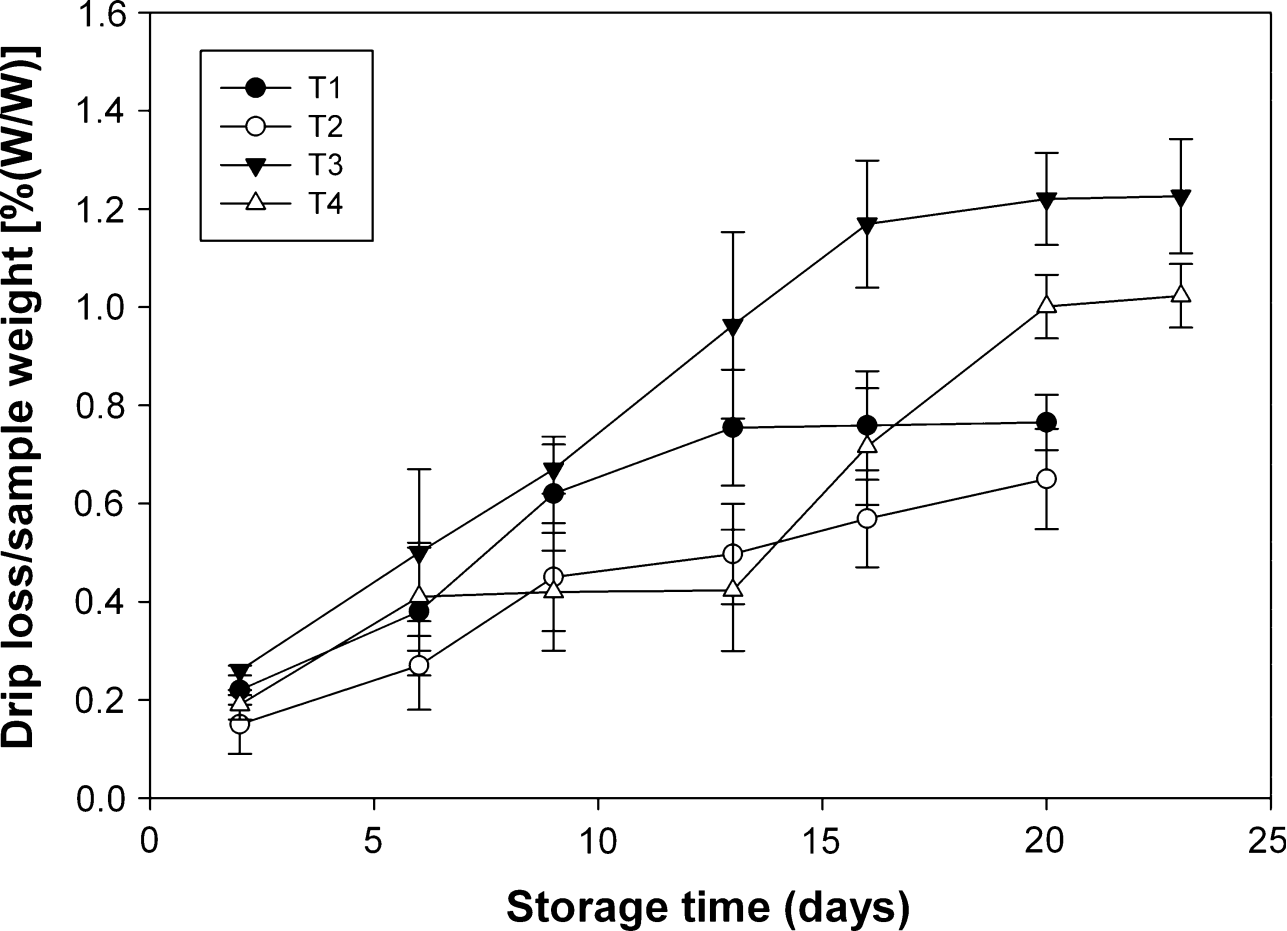
Drip loss in air- and modified atmosphere (MA)-packaged tilapia fillets during storage at 1°C and −1°C. Values (percentage of initial sample weight) are given as mean±SEM or SD (average values for packs, *n* = 3).

The pH values measured were similar and slightly higher in air- than MA-packaged tilapia fillets (Table 5), as could be expected due to the acidic effect of dissolved CO_2_ (Sivertsvik et al. 2002). This partly explains the bacteriostatic effects of CO_2_ in MAP (Huss 1995). Overall, storage time and atmosphere interaction did not affect significantly pH values of fillets (*P* > 0.05). Similarly Reddy et al. (1995) did not find significant differences in pH between packing atmospheres (air and 75% CO_2_/25% N_2_) during storage of tilapia fillets at abused and refrigerated temperature, but their higher temperature (4–16°C) is expected to have caused a lesser CO_2_ quantity to dissolve. The slight difference in pH observed contributed significantly to drip loss reported among the groups (Fig. 5). Postmortem pH according to Huss (1988) is the most significant factor influencing the texture of the meat and the degree of “gaping,” that is, the rapture of the connective tissue. One of the reasons for this is that even minor changes in pH drastically affect the properties of the connective tissue directly affecting the water-holding capacity of the fish muscle and in turn the drip loss.

Higher drip was observed in MA-packaged groups with T3 recording higher values that were significantly different from T2 (*P* < 0.05) on day 13, 16, and 20 for T2 and T1 (Fig. 5). With respect to packaging atmosphere, higher drip was recorded with storage at refrigeration (1°C) than superchilling (−1°C). This agrees with Sivertsvik et al. (2003) who reported that superchilled storage leads to less exudation probably due to high water-holding capacity at low temperature. According to FAO (1996), drip loss is defined as the nutrients from and value of fish, which is potentially available for human consumption, but fails to be consumed or sold as products. Drip during transportation of product from source to final consumer or storage may result to weight below legally allowed tolerance even if it is wholesome and fit for consumption. It is therefore evidenced that air-packaged tilapia fillets stored at −1°C (T2) showed better quality related to analyzed physical properties.

### TVB-N and TMA

TVB-N, TMA, and other volatile amines are commonly used as indicator for fish deterioration in fresh and lightly preserved seafood (Kilcast and Subramaniam 2000). Volatile bases result from degradation of proteins and nonprotein nitrogenous compounds. The study depicted slow TVB-N accumulation in all sample groups (<25 mg N/100 g) during storage at 1°C and −1°C (data not shown). Nonetheless at the end of storage time (day 20), T1 was different (21 mg N/100 g) to other groups but within concentration of 30 mg/100 g, above which fish is considered unfit for consumption. This is because changes in some of volatile nitrogen bases are influenced most importantly by microorganisms which were >log 8 CFU g^−1^ in T1 on day 20. Ababuocha et al. (1996) showed sardines accumulated TVB-N faster when stored at ambient temperature than on ice probably because of increased growth and activity of spoilage and mesophilic bacteria.

The amount of TMA in tilapia fillets during storage time was very low (<1 mg/100 g) and to some extent not detected in most groups. This is probably because TMAO is virtually absent from freshwater species and terrestrial organisms (Hebard et al. 1982). Conversely, some studies have reported TMA in tilapia fish (Reddy et al. 1995; Waliszewski and Avalos 2001). According to Waliszewski and Avalos (2001), the presence of TMA is attributed to marine fish meal fed on tilapia.

### Shelf life

Descriptive analysis can be useful in shelf life studies, as it provides information about maximum shelf life (Bonilla et al. 2007; Cyprian et al. 2008). The results from descriptive analysis and microbiological analysis indicated that air-packaged tilapia fillets had a shelf life of 13–15 days when stored at 1°C (T1) in contrast to about 20 days when stored at −1°C (T2). On the other hand, MA-packaged fillets on day 23 were within consumption limits as evaluated with descriptive analysis (score <20 for spoilage attributes) and microbiologically suitable for consumption. Nevertheless, the MA-packaged groups were unwanted around day 6 based on appearance of raw fillets caused by changes in color as well as mucus and texture. This is because color of the fresh meat is an important indicator of tilapia quality and a major factor in influencing retail purchase decisions. Reddy et al. (1995) reported MA-packaged (75% CO_2_/25 N_2_) tilapia fillets stored at 4°C to have a shelf life of >25 days based on microbial evaluation, which is longer than the findings from this study despite storage at low temperature. This could be attributed mainly to emphasis placed on appearance in the current study.

## Conclusions

MAO performed an effective synergy with superchilling in extending microbiological quality of fillets as the increase in viable cell counts was suppressed compared with counterparts refrigerated and air packaged. Conversely, MA-packaging negatively influenced color, drip loss, and texture of products which limited its application in extending the shelf life of tilapia fillets. Based on sensory evaluation of cooked tilapia and microbial counts, the maximum storage time was 13–15 days for air-packaged fillets stored at 1°C (T1) and 21 days for those superchilled (T2).

Superchilled, air-packaged samples (T2) retained freshness color characteristic longer, recorded less drip loss, and suppressed microbial growth compared with refrigerated air-packaged samples (T1). Therefore, primarily based on sensory evaluation, but also on physical properties and microbiological and chemical data, it can be concluded that optimal condition for storage of fresh tilapia fillets was air packaged, the suitable temperature being −1°C, and the storage time 20 days. It was observed in this study that both TVB-N and TMA did not provide a useful index of shelf life to reflect spoilage in tilapia fillets during storage.
